# Effects of Sodium Pyruvate on Vanadyl Sulphate-Induced Reactive Species Generation and Mitochondrial Destabilisation in CHO-K1 Cells

**DOI:** 10.3390/antiox11050909

**Published:** 2022-05-05

**Authors:** Iwona Zwolak, Ewa Wnuk

**Affiliations:** Centre for Interdisciplinary Research, The John Paul II Catholic University of Lublin, Konstantynów Ave 1J, 20-708 Lublin, Poland; ewa.wnuk@kul.pl

**Keywords:** vanadyl sulphate, pyruvate, oxidative stress markers, mitochondrial membrane potential, heavy metals

## Abstract

Vanadium is ranked as one of the world’s critical metals considered important for economic growth with wide use in the steel industry. However, its production, applications, and emissions related to the combustion of vanadium-containing fuels are known to cause harm to the environment and human health. Pyruvate, i.e., a glucose metabolite, has been postulated as a compound with multiple cytoprotective properties, including antioxidant and anti-inflammatory effects. The aim of the present study was to examine the antioxidant potential of sodium pyruvate (4.5 mM) in vanadyl sulphate (VOSO_4_)-exposed CHO-K1 cells. Dichloro-dihydro-fluorescein diacetate and dihydrorhodamine 123 staining were performed to measure total and mitochondrial generation of reactive oxygen species (ROS), respectively. Furthermore, mitochondrial damage was investigated using MitoTell orange and JC-10 staining assays. We demonstrated that VOSO_4_ alone induced a significant rise in ROS starting from 1 h to 3 h after the treatment. Additionally, after 24 and 48 h of exposure, VOSO_4_ elicited both extensive hyperpolarisation and depolarisation of the mitochondrial membrane potential (MMP). The two-way ANOVA analysis of the results showed that, through antagonistic interaction, pyruvate prevented VOSO_4_-induced total ROS generation, which could be observed at the 3 h time point. In addition, through the independent action and antagonistic interaction with VOSO_4_, pyruvate provided a pronounced protective effect against VOSO_4_-mediated mitochondrial toxicity at 24-h exposure, i.e., prevention of VOSO_4_-induced hyperpolarisation and depolarisation of MMP. In conclusion, we found that pyruvate exerted cytoprotective effects against vanadium-induced toxicity at least in part by decreasing ROS generation and preserving mitochondrial functions

## 1. Introduction

Vanadium (V) is a metal commonly used in many industries, particularly in the production of steel alloys to improve their hardness and strength, and as an industrial catalyst for the production of sulphuric acid. It can also be found in ceramics and glass industries. The latest application of V is the production of V redox batteries for the storage of power from renewable energy sources [1,2]. V is considered an environmental pollutant released principally by the production and combustion of coal and petroleum and mining of V ores [3]. It has been estimated that the anthropogenic input of V to the atmosphere may be 1.7 times greater than the V input from natural sources [4]. The sources of human exposure to V mostly include occupational settings, such as mining and V processing, and local environmental exposures in regions contaminated with V as a result of metallurgic/mining activities and fuel combustion [5]. Accordingly, measurement of the concentration of V in the serum of workers exposed to vanadium pentoxide showed an average concentration of 7.73 μg V/L (in the unexposed control group 3.43 μg/L) but it did not induce genotoxic changes in leukocytes of the exposed group [6]. On the contrary, genotoxic changes (such as micronucleus induction) in blood cells were observed in workers exposed to V dust showing a significantly elevated concentration of V in the plasma (2.2 μg/L, control: 0.3 μg/L) [7]. In the Mt Etna volcanic area an increased incidence of thyroid cancer, and an increased concentration of vanadium in the urine equal to 0.16 μg/g creatinine (against the value of 0.02 μg/g creatinine in non-volcanic areas) was reported [8]. Other reported effects of occupational or environmental exposure to vanadium also included altered neurobehavioral functions [9], higher risk of cardiovascular and respiratory hospitalisations in USA populations [10], impaired foetal growth, and higher risk of preterm and early-term birth, as reported in Chinese studies [11,12].

Oxidative stress has been reported as one of the major mechanisms involved in the toxic effects of V (reviewed by Ścibior and Kurus [13]). For example, results of in vivo studies indicated that V induced lipid peroxidation and hydrogen peroxide (H_2_O_2_) generation and decreased the activities of antioxidant enzymes in the brain of mice [14]. Another study found that V (as sodium metavanadate (NaVO_3_)) induced reactive oxygen species (ROS) generation and decreased the level of reduced glutathione (GSH) in the brain of rats [15]. Samira et al. [16] demonstrated that V (as VOSO_4_) induced oxidative damage in the liver of nondiabetic rats. The direct mechanism implicated in ROS production by V includes interconversion between V^4+^ and V^5+^ by the action of cellular oxidants and antioxidants in the cytoplasm during which toxic free radicals, i.e., hydroxyl radical (^•^OH) and superoxide anion (O_2_^•^^−^), are produced. In addition, the deleterious effects of V on mitochondria and V-dependent stimulation of mitochondrial oxidative stress may also contribute to V-induced ROS generation [17].

Pharmacological treatment of vanadium poisoning, like that of other metals, includes the use of chelating agents. However, as studies show, the use of chelators, including calcium disodium ethylenediaminetetraacetate (CaNa2EDTA) in calves [18] or Tiron (sodium 4,5-dihydroxybenzene-l,3-disulfonate) in laboratory animals [19], provided only limited protection against vanadium. Moreover, it is also known that chelating agents, due to their narrow therapeutic range, can give rise to unfavorable side effects. Therefore, the use of antioxidants is suggested as an adjunct method in the treatment of metal intoxication [20]. Recent experimental studies have shown that pyruvate, thanks to its direct or indirect antioxidant properties, has the potential to treat the toxicity of cadmium, aluminum and zinc [21,22,23] and vanadium [17]. Moreover, preliminary clinical studies, e.g., in patients with heart failure [24] or mitochondrial diseases [25] have demonstrated safety and high tolerance of pyruvate with few side effects.

Pyruvic acid is a small α-keto acid molecule. In the form of pyruvate, it is the final product of the glycolysis pathway. It is subsequently reduced to lactate (in the cytoplasm) or converted to acetyl-CoA (in mitochondria) to enter the tricarboxylic acid cycle [26]. However, in addition to being an energy-supplying metabolic fuel, pyruvate has been reported to display potent antioxidant, anti-inflammatory, and antiapoptotic properties [27,28]. Researchers have reported many beneficial effects of pyruvate, such as decreased incidence of type 1 diabetes in mice [29], protection against pancreatic damage in a rat model [30] and beneficial actions against cardiovascular diseases (reviewed by Mallet et al. [31]). In addition, as shown by Ramos-Ibeas et al. [32], pyruvate (as sodium pyruvate) was the most protective antioxidant among such known antioxidant compounds as N-acetylcysteine (NAC), Trolox (a water-soluble vitamin E analogue), and selenium (as sodium selenite) against oxidative stress in human fibroblasts and embryonic stem cells.

The Chinese hamster ovary (CHO)-K1 cell line due to its uniform properties and high proliferative capability is often used as a model of mammalian cells to study and characterize the toxic effects of various xenobiotics, including mycotoxins [33], nanoparticles [34], ultrafine particles [35] as well as heavy metals. With regard to the study of metals, the CHO-K1 cells have been proven to be a useful in vitro model, e.g., in the screening of the cytotoxicity of inorganic heavy metal compounds [36,37], in the cytotoxicity assessment of organic transition metal complexes (Mn, Ni and Cu) [38] and also in the evaluation of the influence of metals (e.g., Cd, Hg, Pb) on the antioxidant capacity of cells [36] and in the evaluation of the genotoxic activity of manganese and aluminum [39]. Additionally, the biological effects of vanadium are often assessed with CHO-K1 cells. These studies concerned, among others, the genotoxic potential of vanadium [40,41], the comparison of the pro-oxidative abilities of inorganic and organic vanadium salts [42], the cytotoxicity assessment of vanadium-based anticancer agents [43] and the insulin-like properties of vanadium [44]. Therefore, the CHO-K1 cells were considered to be a suitable in vitro model to study the adverse effects of vanadium.

In our lab, we have previously shown that VOSO_4_-induced cytotoxicity and lipid peroxidation were partly prevented by pyruvate in CHO-K1 cells [28]. However, the antioxidant mechanism underlying the beneficial actions of pyruvate against vanadium is still obscure. The present work was undertaken to contribute to the mechanisms underlying this antagonistic interaction of vanadium and pyruvate under the same experimental conditions. Considering the following points: (1) ROS generation is an important upstream event in V-mediated cell damage, (2) V-induced oxidative stress is related to prooxidant effects of V on mitochondria [45], (3) mitochondrial ROS generation triggers changes in mitochondrial membrane potential (MMP), (4) pyruvate has been shown to induce beneficial effects on mitochondria [21,32], in the current study, we investigated the effects of pyruvate against V-induced ROS generation and V-triggered MMP disturbances.

## 2. Materials and Methods

### 2.1. Reagents

Dulbecco’s modified Eagle’s medium Hams F-12 (DMEM)/F12 1:1 (cat. No. D8437), fetal bovine serum (FBS, Cat No. F9665), antibiotic antimycotic solution (100×) (cat. No. A5955), vanadyl sulphate hydrate (VOSO_4_∙xH_2_O, cat. No. 204862), sodium pyruvate solution (100 mM, cat No. S8636), In Vitro Toxicology Assay Kit, Resazurin based (cat. No. TOX8), dihydrorhodamine 123 (DHR 123, cat. No. D1054), and Mitochondrion Potential Membrane Kits, i.e., MitoTell orange (cat. No. MAK147) and JC-10 dye (cat. No. MAK159) were purchased from Sigma-Aldrich (St. Louis, MI, USA). The OxiSelect™ Intracellular ROS Assay Kit (green fluorescence, cat. No. STA-844) was purchased from Cell Biolabs (San Diego, CA, USA). Trypsin solution (0.25%) was supplied by Biomed, Lublin, Poland.

VOSO_4_∙xH_2_O (assuming hydration of five molecules) was dissolved in deionised water to a final concentration of 10 mM/l stock solution (light blue in colour). The stock solution was prepared freshly every time just before experiments involving exposure of the cells to VOSO_4_.

### 2.2. Cell Culture

The CHO-K1 cell line (Chinese hamster ovary cell line K1) was a generous gift from Drs. W. Trybus and E. Trybus (Department of Medical Biology, Institute of Biology, Jan Kochanowski University in Kielce, Poland). The CHO-K1 cells were cultured in DMEM/F12 1:1 containing 5% FBS, 100 U/mL penicillin, 100 mg/mL streptomycin, and 0.25 µg/mL amphotericin B in a humidified incubator (CO_2_ incubator HERAcell 150i, Thermo Electron LED GmbH, Langenselbold, Germany) at 37 °C and 5% CO_2_. The cultures were passaged twice a week using a 0.25% trypsin solution. The cells were observed using a phase-contrast microscope (Olympus, model IX73, Tokyo, Japan). All procedures necessitating sterile conditions were carried out using a laminar flow cabinet (Herasafe laminar flow cabinet, model KS, Thermo Electron LED GmbH, Langenselbold, Germany).

### 2.3. Dose Selection

The concentration of VOSO_4_ (100 μM) was chosen on the basis of our previous reports, which showed the cytotoxicity of 100 μM VOSO_4_ in CHO-K1 cells [28,46]. We also considered other studies which assessed toxicity mechanisms of compounds with vanadium in the +4 and +5 oxidation states (VOSO_4_ and NaVO_3_) using similar concentrations in the A549 cell line [47], human lymphocytes and HeLa cells [48], and isolated rat mitochondria [45].

The concentration of pyruvate (4.5 mM) was selected on the basis of our previous research with this compound [28] and our concentration range-finding experiment, which showed that the 4–4.5 mM concentration from the 2–8 mM range induced the most optimal cell protection, as assessed by morphological analysis and cell viability assay (Figures S1 and S2). Of note, sodium pyruvate at similar doses as this used in our study showed cytoprotective effects in hippocampal HT-22 cells [21], rat cerebellar granular cell cultures [49], human neuroblastoma SK-N-SH cells [50], and murine fibroblasts (NIH3T3) and myoblasts (C2C12) [51].

### 2.4. Determination of Cytotoxicity Using the Resazurin Assay

The principle of the resazurin-based assay is the reduction of blue resazurin dye to red resorufin by mitochondria in living cells. The amount of resazurin that is not reduced to resorufin is proportional to the number of injured cells. The absorbance of resazurin is read at 600 nm and decreases proportionally to the number of living cells.

For the assays, the CHO-K1 cells (1 × 10^4^ cells/well) were seeded into clear 96-well plates in DMEM/F12 (containing 5% FBS) and maintained at 37 °C in 5% CO_2_. After 24 h of plating, the cell culture medium was removed and the cells were treated with DMEM/F12–5% FBS (100 µL) containing VOSO_4_ (100 µM) and pyruvate (4.5 mM). Following 24-h incubation (at 37 °C in 5% CO_2_), the medium with the tested compounds was replaced with DMEM/F12 (without FBS) and 10 μL of resazurin dye was added to each well. The absorbance of resazurin was red at 600 nm (690 nm background) in a microplate reader (Synergy 2, BioTek Instruments, Inc., Winooski, VT, USA). Two independent experiments were performed with six wells per treatment condition. The data from the resazurin assay are expressed as the percentage of control cells. In this assay, cytotoxicity (cell damage) is indicated by an increase in percentage values, compared to the control cells.

### 2.5. Determination of Total and Mitochondrial ROS Production Using DCFH-DA and DHR123 Dyes

The general ROS production was measured using an OxisSelect™ Intracellular ROS assay kit (green fluorescence), following the instruction provided by the manufacturer. The assay uses the cell-permeable fluorogenic probe, 2′,7′-dichlorodihydrofluorescein diacetate (DCFH-DA). The DCFH-DA dye diffuses passively into cells and is hydrolysed by intracellular esterases to give non-fluorescent 2′,7′-dichlorodihydrofluorescein (DCFH), which is trapped within the cells. In the presence of ROS, DCFH is oxidised to highly fluorescent 2′,7′-dichlorofluorescein (DCF). Dihydrorhodamine 123 (DHR 123) was the probe used in this study to assess mitochondrial ROS. DHR 123 diffuses freely into cells, where it is oxidised by ROS into fluorescent cationic rhodamine 123 accumulating in mitochondria potentiometrically.

For the assays, the CHO-K1 cells (1 × 10^4^ cells/well) were seeded into black 96-well plates in DMEM/F12 (containing 5% FBS) and maintained at 37 °C in 5% CO_2_. After 24 h from plating, the cell culture medium was removed and the cells were preloaded with 10 µM DCFH-DA or 25 μM DHR123 for 20 min at 37 °C. Then, the cells were carefully washed once with serum free DMEM-F12 to remove the unbounded DCFH-DA or DHR123 dye. Next, the cells were treated with DMEM/F12–5% FBS (100 µL) containing VOSO_4_ (100 µM) and pyruvate (4.5 mM). Following 1-, 2- and 3-h incubation of the cells in the medium with the tested compounds (at 37 °C in 5% CO_2_), the fluorescence of dichlorofluorescein (DCF) or rhodamine 123 (RH-123) was analysed in a microplate reader (Synergy 2, BioTek Instruments, Inc., Winooski, VT, USA) using excitation and emission wavelengths of 485 nm and 528 nm, respectively. The data (ROS levels) were expressed as a percentage of control cells.

### 2.6. Mitochondrial Membrane Potential (MMP) Assessment with MitoTell Orange Dye

The MitoTell^TM^ orange dye exhibits potential-dependent accumulation in mitochondria selectively generating orange fluorescence emission at 590 nm.

For the assays, the CHO-K1 cells (1 × 10^4^ cells/well) were seeded into black 96-well plates and grown for 24 h. Then, the cells were treated with VOSO_4_ (100 µM) in the presence or absence of pyruvate (4.5 mM) for the next 24 h and 48 h, and MMP was determined according to the manufacturers’ instructions.

Accordingly, for the MitoTell orange dye staining, the cell culture medium was removed and 100 μL/well of the dye loading solution was added to each well. The plate was incubated at 37 °C in a humidified atmosphere of 5% CO_2_ for 15 min. Assay buffer B (50 μL) was added to each well and, after 20-min incubation, the fluorescence intensity was recorded in a microplate reader (Synergy 2, BioTek Instruments, Inc., Winooski, VT, USA) using excitation and emission wavelengths of 530 nm and 590 nm, respectively.

### 2.7. Mitochondrial Membrane Potential (MMP) Assessment with JC-10 Dye

The JC-10 dye is concentrated in polarised mitochondria where it forms red fluorescent JC-10 aggregates (590 nm). In apoptotic cells with reduced MMP, JC-10 remains in the cytosol in a monomeric form and emits green fluorescence (530 nm).

For the assays, the CHO-K1 cells (1 × 10^4^ cells/well) were seeded into black 96-well plates and grown for 24 h. Then, the cells were treated with VOSO_4_ (100 µM) in the presence or absence of pyruvate (4.5 mM) for the next 24 h and 48 h, and MMP was determined according to the manufacturers’ instructions.

Accordingly, the JC-10 dye loading solution (50 μL) was added directly to the cell culture medium of the sample and control wells. The plate was incubated in 5% CO_2_ at 37 °C for 30 min and then assay buffer B (50 μL) was added to each well. The fluorescence intensity was recorded in a microplate reader (Synergy 2, BioTek Instruments, Inc., Winooski, VT, USA) using 485 nm/528 nm (ex/em) and 530 nm/590 nm (ex/em) filters.

### 2.8. Statistical Analysis

**Figure 1:**
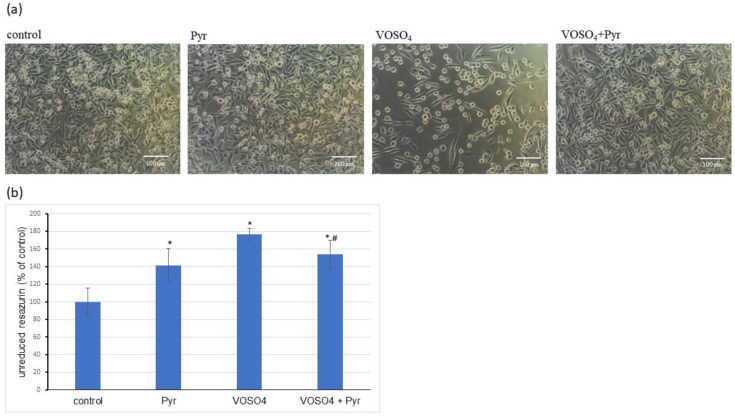
Effects of pyruvate on VOSO_4_-induced cytotoxicity in CHO-K1 cells. The CHO-K1 cells were treated with 100 μM VOSO_4_ in the presence or absence of sodium pyruvate (Pyr) (4.5 mM) for 24 h. (**a**) Morphology of CHO-K1 cells under the phase-contrast microscope (scale bar = 100 μM), (**b**) resazurin-based cytotoxicity assay. Data represent the mean ± SD of two distinct experiments each performed with sixplicate determinations of each data point, * *p* < 0.001, versus the control group, # *p* < 0.05 versus the VOSO_4_-treated group. Data were analysed by Student’s *t*-test.

**Figure 2:**
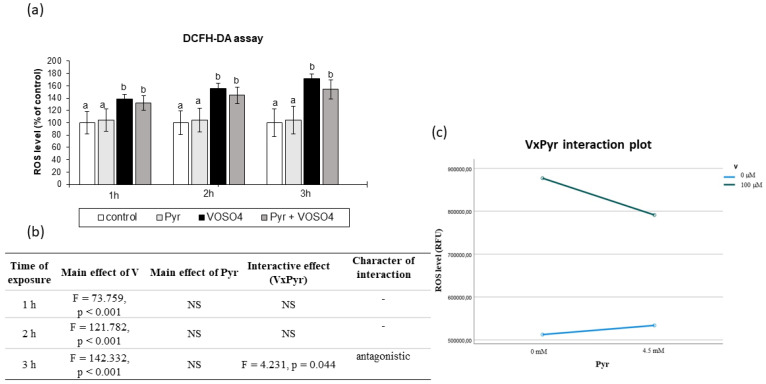
Effects of vanadium and/or pyruvate on ROS accumulation in CHO-K1 cells detected by the DCFHDA assay. The H2DCFDA-preloaded cells were incubated in DMEM/F12 medium without (control) or with 100 μM VOSO_4_ in the presence or absence of sodium pyruvate (Pyr) (4.5 mM) for 1, 2 and 3 h. (**a**) Intracellular accumulation of ROS on the basis of DCF fluorescence emissions expressed in percentage. Data represent the mean ± SD of quadruplicate determinations of three distinct experiments. Means at the same time point followed by a common letter are not significantly different. Data were analysed by one-way ANOVA followed by Tukey’s post hoc test (**b**) Results of the two-way ANOVA analysis of DCF fluorescence emissions after 1-, 2-, and 3-h exposure to V (as VOSO_4_) and Pyr (pyruvate); NS: no significant effect. (**c**) Interaction graph illustrating antagonistic interaction between V and Pyr for the intracellular ROS level after 3-h incubation.

**Figure 3:**
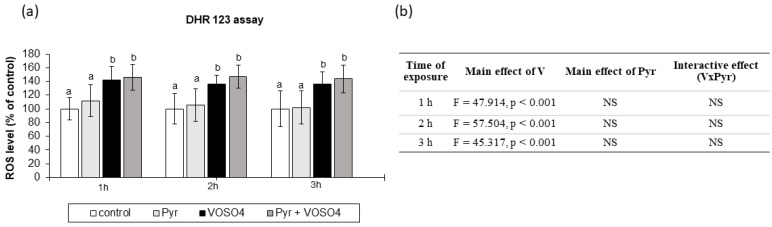
Effects of vanadium and/or pyruvate on ROS accumulation in CHO-K1 cells detected by the DHR123 assay. The DHR123-preloaded cells were incubated in DMEM/F12 medium without (control) or with 100 μM VOSO_4_ in the presence or absence of sodium pyruvate (Pyr) (4.5 mM) for 1, 2, and 3 h. (**a**) Intracellular accumulation of ROS on the basis of RH-123 fluorescence emissions expressed in percentage. Data represent the mean ± SD of quadruplicate determinations of 3 distinct experiments. Means at the same time point followed by a common letter are not significantly different. Data were analysed by one-way ANOVA followed by Tukey’s post-hoc test. (**b**) Results of the two-way ANOVA analysis of RH-123 fluorescence emissions after 1-, 2-, and 3-h exposure to V (as VOSO_4_) and Pyr (pyruvate); NS: no significant effect.

**Figure 4:**
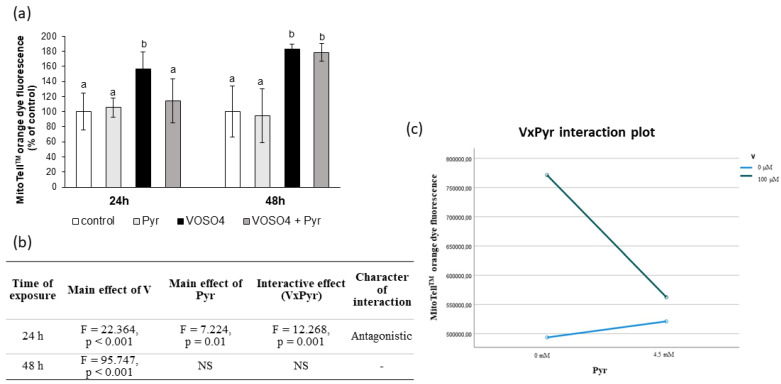
Effects of vanadium and/or pyruvate on MMP in CHO-K1 cells detected with the MitoTell staining method. The cells were incubated in DMEM/F12 medium without (control) or with 100 μM VOSO_4_ in the presence or absence of sodium pyruvate (Pyr) (4.5 mM) for 24 h and 48 h. (**a**) MMP on the basis of MitoTell orange dye fluorescence emissions expressed in percentage. Data represent the mean ± SD derived from 3 independent experiments each performed at least in triplicates. Means at the same time point followed by a common letter are not significantly different. Data were analysed by one-way ANOVA followed by Tukey’s post hoc test, (**b**) Results of the two-way ANOVA analysis of MitoTell orange fluorescence emissions after 24- and 48-h exposure to V (as VOSO_4_) and Pyr (pyruvate); NS: no significant effect; (**c**) interaction graph illustrating antagonistic interaction between V and Pyr for MMP after 24-h incubation.

**Figure 5:**
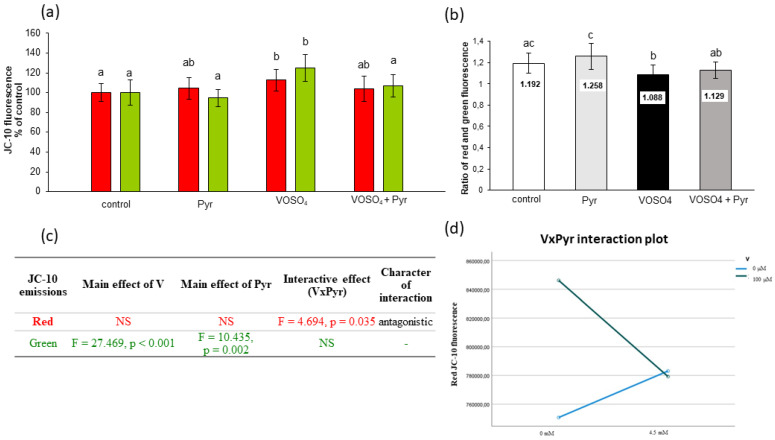
Effects of vanadium and/or pyruvate on MMP in CHO-K1 cells detected with the JC-10 staining method. The cells were incubated in DMEM/F12 medium without (control) or with 100 μM VOSO_4_ in the presence or absence of sodium pyruvate (Pyr) (4.5 mM) for 24 h. (**a**) Red and green JC-10 fluorescence emissions expressed in percentage, and (**b**) red/green fluorescence intensity ratio. Data represent the mean ± SD derived from 3 independent experiments each performed at least in triplicates. Means within the same color followed by a common letter are not significantly different. Data were analysed by one-way ANOVA followed by Tukey’s post hoc test; (**c**) Results of the two-way ANOVA analysis of red and green JC-10 fluorescence emissions after 24-h exposure to V (as VOSO_4_) and Pyr (pyruvate); NS: no significant effect; (**d**) interaction graph illustrating antagonistic interaction between V and Pyr for red JC-10 fluorescence emissions after 24-h incubation.

**Figure 6:**
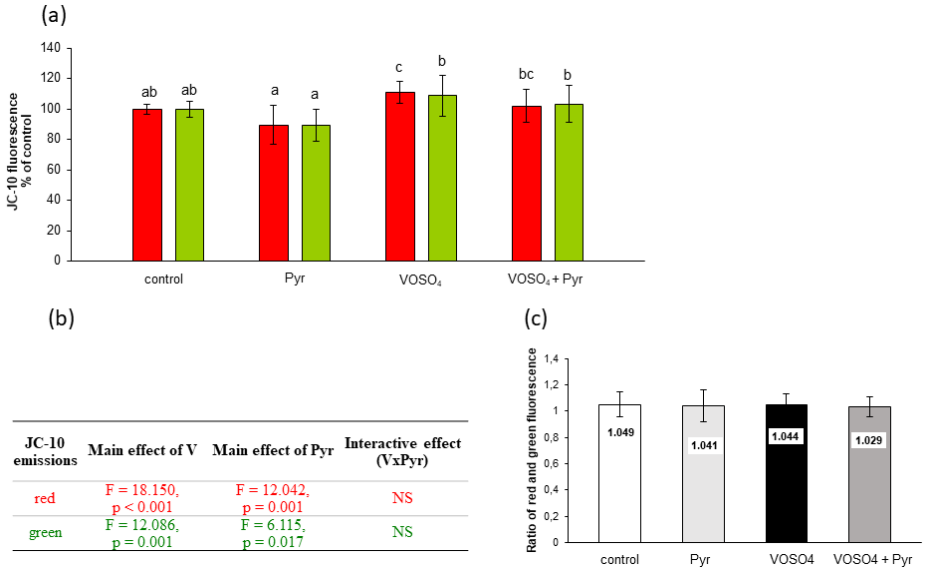
Effects of vanadium and/or pyruvate on MMP in CHO-K1 cells detected with the JC-10 staining method. The cells were incubated in DMEM/F12 medium without (control) or with 100 μM VOSO_4_ in the presence or absence of sodium pyruvate (Pyr) (4.5 mM) for 48 h. (**a**) Red and green JC-10 fluorescence emissions expressed in percentage. Data represent the mean ± SD derived from 3 independent experiments each performed at least in triplicates. Means within the same color followed by a common letter are not significantly different. Data were analysed by one-way ANOVA followed by Dunnett’s T3 test; (**b**) Results of the two-way ANOVA analysis of red and green JC-10 fluorescence emissions after 48-h exposure to V (as VOSO_4_) and Pyr (pyruvate), NS: no significant effect; (**c**) red/green fluorescence intensity ratio.

All quantification results were obtained from at least two-three independent experiments to confirm data reproducibility and reliability. Data were analysed using the Statistical Package for the Social Sciences (IBM Corp. Released 2020. IBM SPSS Statistics for Windows, Version 27.0. Armonk, NY: IBM Corp). To meet the assumptions of ANOVA, the data were checked for outliers using Tukey’s fence. The normal distribution was checked by Shapiro–Wilk or Kolmogorov–Smirnov tests. The homogeneity of variances was analysed with Levene’s test. A one-way ANOVA analysis, Tukey’s post hoc test (equal variances assumed), Dunnett’s T3 test (unequal variances assumed) (Figures 2–6) and Student’s *t*-test (Figure 1) were performed to assess the differences between the four treatment groups (control, pyruvate, VOSO_4_, and pyruvate + VOSO_4_). A two-way ANOVA with Bonferroni post test was performed to detect significant main effects of pyruvate and VOSO_4_ and interactive pyruvate × VOSO_4_ effects on the measured parameters. Two-way ANOVA was used to increase the statistical power, compared to using one-way ANOVA for the detection of significant differences between the treatment groups and identification of the interaction between pyruvate and VOSO_4_ [52]. A significance level of *p* < 0.05 was used for all tests.

## 3. Results

### 3.1. Cytotoxicity Assessment

We first confirmed by morphological observations that the deterioration in the CHO-K1 cell morphology caused by the 24-h exposure to 100 μM VOSO_4_ was inhibited by the presence of 4.5 mM sodium pyruvate (Figure 1a). As determined by the resazurin assay, the cytotoxicity induced by 100 μM VOSO_4_ was significantly reduced in the presence of 4.5 mM pyruvate (Figure 1b). Interestingly, in the test with resazurin, a significant increase in cytotoxicity was observed after the 24-h incubation of CHO-K1 cells with 4.5 mM sodium pyruvate alone. This result was unexpected because, in our previous studies, incubation of the same cells with the aforementioned dose of sodium pyruvate did not cause changes in cell viability as measured by this test [28]. Nevertheless, as mentioned above, the tested dose of pyruvate was protective against the cytotoxicity of VOSO_4_ as well as protective against most of the other parameters tested (described later in the results). Moreover, as can be seen from the photos (Figure 1a), the cells treated with pyruvate alone did not differ from the controls. Additionally, other parameters, such as ROS or MMP (Mitochondrial Membrane Potential) levels were not changed in pyruvate control as shown in Figure 2, Figure 3, Figure 4, Figure 5 and Figure 6. Therefore, we suspect that the inhibition of resazurin reduction in cells treated with sodium pyruvate alone was not related to pyruvate toxicity but was a kind of artifact.

### 3.2. ROS Generation Assessed by DCFH-DA Staining

We then examined the intracellular production of ROS in the CHO-K1 cells using DCFH-DA staining after the 1-, 2-, and 3-h exposure to 100 μM VOSO_4_ in the absence or presence of 4.5 mM sodium pyruvate. As presented in Figure 2a, the ROS level in the CHO-K1 cells after the incubation with 100 μM VOSO_4_ for 1, 2 and 3 h were significantly increased, compared to the control. The ROS production in the cells treated with 100 μM VOSO_4_ + 4.5 mM pyruvate was non-significantly reduced (by 16.8%), compared to the VOSO_4_-exposed cells after a 3-h exposure. As demonstrated by two-way ANOVA, this decrease was caused by the antagonistic interaction between VOSO_4_ and pyruvate (Figure 2b,c). The cells treated only with pyruvate for 1, 2, and 3 h had a similar intracellular ROS level to that in the control.

### 3.3. ROS Generation Measured by DHR123 Staining

We also examined the intracellular ROS production in the CHO-K1 cells using the DHR123 method. As shown in Figure 3a, the ROS levels were significantly increased in cells exposed to VOSO_4_, compared to the control at 1, 2, and 3 h. The simultaneous treatment with pyruvate + VOSO_4_ did not change the ROS production, compared to the VOSO_4_ only exposed cells at any exposure time point. The cells treated only with pyruvate for 1, 2, and 3 h had a similar intracellular ROS level to that in the control. As presented in Figure 3b, the two-way ANOVA analysis revealed that the ROS generation was significantly affected only by the main effect of VOSO_4_. However, there was no significant pyruvate effect and no VOSO_4_ x pyruvate interaction on the ROS levels at all the exposure time points.

### 3.4. Mitochondrial Membrane Potential Assessed by MitoTell Orange Staining

Further, we assessed the mitochondrial membrane potential (MMP) in the CHO-K1 cells using the MitoTell orange dye. As shown in Figure 4a, the 24-h exposure of the cells to VOSO_4_ significantly increased (by 56.2%) orange fluorescence intensity, compared to the control. As compared to the VOSO_4_-exposed cells, the fluorescence intensity emitted by the VOSO_4_ + pyruvate co-treated cells was highly significantly decreased (by 42.3%, *p* = 0.001), and this decrease was related to a significant interactive effect between VOSO_4_ and pyruvate (Figure 4b,c). In turn, the 48-h co-treatment with pyruvate + VOSO_4_ did not change the orange fluorescence intensity, compared to the VOSO_4_ only exposed cells.

### 3.5. Mitochondrial Membrane Potential Assessed by JC-10 Staining

MMP was also assessed with the use of JC-10 fluorescence dye to support the MitoTell orange dye assay. The JC-10 dye allows measurement of MMP by evaluating red and green fluorescence emissions or calculating the red:green ratio. As presented in Figure 5a, the CHO-K1 cells exposed to VOSO_4_ for 24 h had significantly increased red (by 12.7%) and green (by 24.8%) fluorescence emissions in comparison with the control. In the pyruvate + VOSO_4_ cotreated cells, the red and green fluorescence decreased by 8.9% and 17.6%, respectively, compared to the VOSO_4_ only treated cells (Figure 5a). Inline, the calculated ratio of red:green fluorescence showed a statistically significant reduction in the VOSO_4_-exposed cells compared to the control (Figure 5b). In the cells co-exposed to VOSO_4_ + pyruvate, the red:green fluorescence ratio showed non-significant changes.

As demonstrated by the two-way ANOVA, the decrease in red fluorescence in pyruvate + VOSO_4_ cotreated cells was related to the antagonistic VOSO_4_xPyr interaction (Figure 5c,d). The decrease in green fluorescence intensities in the pyruvate + VOSO_4_ cells was influenced only by the main effect of pyruvate (Figure 5c).

Figure 6 shows the assessment of MMP by JC-10 fluorescence in CHO-K1 cells incubated with 100 μM VOSO_4_ and/or 4.5 mM sodium pyruvate for 48 h. As presented in Figure 6a, the red JC-10 fluorescence in the VOSO_4_-exposed cells showed a significant increase (by 10.8%) in comparison with the control. In turn, the red fluorescence value in the pyruvate + VOSO_4_ cells was only slightly and non-significantly decreased (by 8.8%) compared to the VOSO_4_-treated cells. This decrease was related to the main effect of pyruvate, as suggested by the two-way ANOVA (Figure 6b). The two-way ANOVA also revealed that there was a significant effect of 100 μM VOSO_4_ alone on the green JC-10 fluorescence. There were no significant differences in the red:green ratios between the treatment groups (Figure 6c).

## 4. Discussion

In the current article, we present the results of the first study on the role of ROS and mitochondria in the protective mechanism of pyruvate against VOSO_4_-induced cytotoxicity in CHO-K1 cells. Using four different fluorescent probes, including DCFH-DA and JC-10 dyes, we have shown that VOSO_4_ significantly increased intracellular ROS generation and caused significant changes in the MMP of the CHO-K1 cells, compared to the control cells. In our study, pyruvate applied as sodium pyruvate partially inhibited VOSO_4_-related cytotoxicity, and early ROS generation and largely protected mitochondria from VOSO_4_-induced alterations.

In accordance with our previous study [28], the cytotoxic effect induced by high VOSO_4_ concentration (100 μM) in CHO-K1 cells was successfully prevented by simultaneous treatment with 4.5 mM pyruvate. This was confirmed by both the cell morphology analysis and the resazurin bioassay (Figure 1a). Because the resazurin test assesses cell viability on the basis of the metabolic activity of the mitochondria [53], it could be argued that the functioning of the mitochondria, which is closely related to the production of ROS, may play a key role in the antagonistic interaction between vanadium and pyruvate as described later in this section.

In many in vitro and in vivo models, the triggering mechanism for V-induced toxicity was associated with increased ROS generation [47,54,55,56]. Therefore, in this study, the intracellular ROS overproduction induced by VOSO4 was evaluated using the fluorescent redox-sensitive probes, i.e., DCFH-DA and DHR123. To a large extent, the transition of DCFH and DHR123 to fluorescent dyes (DCF and rhodamine 123, respectively) is triggered by similar mechanisms. In short, both dyes are membrane-permeable and readily diffuse into cells whereupon oxidation by free radicals they are trapped within the cells and emit green fluorescence. Both probes can be oxidised by H_2_O_2_ but only in the presence of such a catalyst as horseradish peroxidase, with DCFH being more dependent on HRP than DHR123 [57]. In cell-free systems, both probes have also been shown to be sensitive in the detection of carbonate radical anion (CO_3_^•−^), nitrogen dioxide (NO_2_^•^) [58], and peroxynitrite (ONOO-) [57,59]. There are, however, some differences between DCFH and DHR123. For example, although both DCFH-DA and DHR123 can be oxidised by ONOO^−^ [57,59], it is the DHR123 dye that is regarded as a very sensitive and specific probe for ONOO^−^ detection due to its ability to detect even very low nanomolar concentrations of this oxidant [60].

In our study, the intracellular ROS production was positively affected already at 1 h after treatment with 100 μM VOSO_4_ (as detected in the DCFH-DA and DHR123 assays, Figure 2 and Figure 3), which indicates that the generation of an oxidising environment is an early event in cells exposed to VOSO_4_. The rise in the ROS generation remained significant following the 2- and 3-h VOSO_4_ treatment in both staining methods. The elevation of ROS production in cells upon V exposure is in accordance with other studies. For example, the same vanadium compound (VOSO_4_) at similar doses (100 and 200 μM) caused a significant increase in ROS production and cytotoxicity in CHO-K1 cells in the work of other researchers [42]. Increased levels of superoxide anion (O_2_^•−^), hydrogen peroxide (H_2_O_2_), and hydroxyl radicals (^•^OH) in lavaged alveolar macrophages from mice have been documented by electron spin resonance (ESR) following in vitro V treatment [56]. Other studies reported that enhanced intracellular formation of H_2_O_2_ was the main mechanism responsible for vanadate-induced cellular damage in monkey kidney epithelial Ma104 cells [61] and mouse epidermal JB6 cells [55] since the catalase enzyme, which specifically degrades H_2_O_2_, resulted in cell protection.

Our results showed that the presence of 4.5 mM pyruvate antagonistically interfered with VOSO_4_-mediated ROS generation, as observed at the 3-h time point in the DCFH-DA assay (Figure 2b,c). On the one hand, this shows that, to some extent, pyruvate has the ability to prevent VOSO_4_-induced ROS generation. On the other hand, however, no pyruvate-mediated protection was observed in the DHR123 method at any exposure time point (Figure 3). Since the DHR123 and DCFH-DA assays may differ in their specificity of detection of particular ROS, the lack of beneficial effects of pyruvate in the DHR123 assay may indicate that the ROS detected by this assay (peroxynitrite in particular) may not be involved in the pyruvate-related antioxidative mechanism in our experimental conditions.

In many studies, the strong pro-oxidant effect of V compounds was accompanied by mitochondrial perturbations. For example, early studies have shown that ROS-mediated mitochondrial damage (seen as a drop in MMP) was an essential step in NaVO_3_-induced apoptosis in mouse epidermal JB6 cells [55]. Zhao et al. [45] found that V-related mitochondrial oxidative stress (detected by DCFH-DA staining), which led to V-induced gradual opening of the mitochondrial permeability transition pore (PTP), caused subsequent mitochondrial swelling and MMP collapse. Additionally, our previous results on CHO-K1 cells showed inhibition of mitochondrial enzymatic activity in response to the toxic effects induced by NaVO_3_ and VOSO_4_ [46,53]. Considering the above studies, we measured MMP as a key marker of mitochondrial function and a potential target of ROS generated during the VOSO_4_ exposure. We found that after the 24- and 48-h exposure, VOSO_4_ caused a significant increase in red fluorescence of two potentiometric dyes: MitoTell (Figure 4a) and JC-10 (Figure 5a and Figure 6a), compared to the control CHO-K1 cells. This indicates a high accumulation of Mitotell dye and JC-10 aggregates in mitochondria due to MMP elevation, suggesting hyperpolarisation of the inner mitochondrial membrane in cells exposed to VOSO_4_. Simultaneously, after the 24- and 48-h incubation, there was a significant effect of VOSO_4_ on green fluorescence emitted by JC-10 monomers (Figure 5c and Figure 6b respectively), which appear when depolarised mitochondria fail to retain JC-10 dye. Following the 24-h exposure to VOSO_4_, the rise in green fluorescence exceeded red fluorescence emissions, indicating that there were more cells that had depolarised mitochondria than those with hyperpolarised mitochondria. However, we showed that the cotreatment of CHO-K1 cells with 4.5 mM pyruvate improves the stability of mitochondria in VOSO_4_-exposed cells at the 24 h time point. This was proven by the antagonistic interactive action of pyruvate on VOSO_4_-induced mitochondrial hyperpolarisation, as shown consistently in both assays, i.e., the MitoTell assay (Figure 4b,c) and the JC-10 method (red JC-10 emission, Figure 5c,d). At the 24 h time point (in the JC-10 assay), pyruvate also prevented VOSO_4_-mitochondrial depolarisation.

Based on our research it can, therefore, be concluded that the protective effect of pyruvate on the cytotoxicity of vanadium towards CHO-K1 cells is partly related to the opposite (antagonistic) effect of both compounds on intracellular ROS production and mitochondrial stability. Among vanadium-induced ROS, H_2_O_2_ probably plays a key role in the mechanism of vanadium cytotoxicity [55,61]. In contrast, pyruvate is an effective antioxidant that non-enzymatically reacts with H_2_O_2_, releasing acetate, CO_2_, and H_2_O (first described by Holleman [62]). We suppose that pyruvate, by acting as a H_2_O_2_ scavenger, could partially reduce the vanadium-induced ROS production (observed in DCFH-DA assay) which then may have contributed to the cytoprotective effect of pyruvate. Moreover, our results of the MMP measurements indicate a mitochondrial compartment in the pyruvate-mediated protection against VOSO_4_-induced cytotoxicity. This is a new result for mutual antagonistic relationships between V and exogenous pyruvate, which may be important considering that other researchers have already highlighted the role of mitochondria in the protective actions of this α-ketoacid against many prooxidant insults [32,50,63,64]. The essential role of mitochondria in pyruvate protective actions was earlier demonstrated by Kang et al. [65]. The authors have shown that α-cyano-3-hydroxycinnamate, which is a selective inhibitor of pyruvate mitochondrial uptake, abolished the limited protective effect of pyruvate against H_2_O_2_-induced injury of pulmonary artery endothelial cells in terms of cell viability and nuclear DNA damage [65]. The potential mechanisms of protection of mitochondria against V by exogenous pyruvate may involve the improvement of mitochondrial energisation [64] and pyruvate-mediated increase of mitochondrial reducing capacity [28,66]. On the other hand, however, the pyruvate-mediated actions against vanadium-induced changes in MMP were only non-significant after the 48-h incubation with VOSO_4_, as shown in the JC-10 method (Figure 6a). The much weaker effects of exogenous pyruvate following the prolonged incubation period may have been related to the depletion of pyruvate in the culture medium. Indeed, pyruvate rapidly decomposes in the reaction with hydrogen peroxide and its concentration in culture medium is known to decrease [67].

## 5. Conclusions

In summary, the present study has shown that pyruvate can be a natural cytoprotector against VOSO_4_-mediated toxicity. Using the CHO-K1 cells, we have provided mechanistic evidence that cytoprotective effects were partly mediated by the antagonistic interaction of pyruvate against VOSO_4_-induced early ROS generation. In addition, through independent action and antagonistic interaction with VOSO_4_, pyruvate substantially protected MMP against VOSO_4_-mediated hyperpolarisation and depolarisation and maintained MMP at control levels. Our combined data suggest that pyruvate enhances intrinsic antioxidant and mitochondrial functions of cells during VOSO_4_ exposure.

## Data Availability

The data presented in this study are available in article and supplementary material.

## Supplementary Materials

The following supporting information can be downloaded at: https://www.mdpi.com/article/10.3390/antiox11050909/s1, Figure S1: Effects of pyruvate on VOSO4-induced changes in morphology of CHO-K1 cells under the phase-contrast microscope (bar = 100 μM); Figure S2: Effects of pyruvate on VOSO4-induced cytotoxicity of CHO-K1 cells in the resazurin-based assay.

## Abbreviations

CHO-K1 cells, Chinese hamster ovary cells; DCFH-DA, 2′,7′-dichlorodihydrofluorescein diacetate; DHR123, dihydrorhodamine 123; DMEM, Dulbecco’s modified Eagle’s medium; FBS, foetal bovine serum; GSH, reduced glutathione; H2O2, hydrogen peroxide; MMP, mitochondrial membrane potential; RH-123, rhodamine 123; ROS, reactive oxygen species; V, vanadium; VOSO4, vanadyl sulphate.
