# Supplementary material for: Effects of Sodium Pyruvate on Vanadyl Sulphate-Induced Reactive Species Generation and Mitochondrial Destabilisation in CHO-K1 Cells
Source: Antioxidants (Basel). 2022 May 5;11(5):909. doi: 10.3390/antiox11050909 (PMC9137755; doi:10.3390/antiox11050909)
Supplement: Supplementary file 1 [file antioxidants-11-00909-s001.zip › antioxidants-1679182-supplementary-done.pdf]

antioxidants

Article

Effects of sodium pyruvate on vanadyl sulphate-induced reactive species generation and mitochondrial destabilisation in CHO-K1 cells

Iwona Zwolak * and Ewa Wnuk

Centre for Interdisciplinary Research, The John Paul II Catholic University of Lublin, Konstantynów Ave 1J, 20-708 Lublin, Poland; ewa.wnuk@kul.pl

* Correspondence: iwona.zwolak@kul.pl; Tel.: +48-814-545-618; Fax: +48-814-454-611

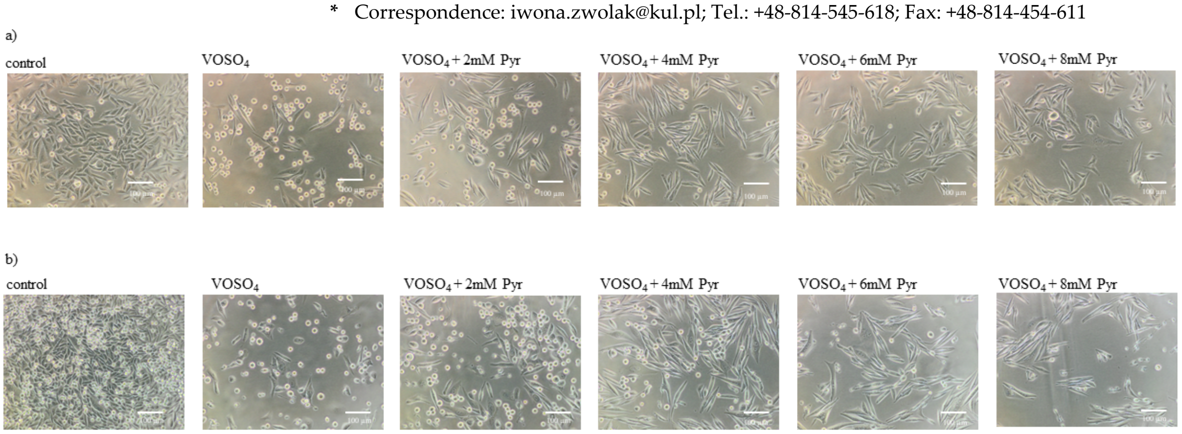

Figure S1. Effects of pyruvate on $VOSO_4$-induced changes in morphology of CHO-K1 cells under the phase-contrast microscope (bar=100 µM). The CHO-K1 cells were treated with 100 µM $VOSO_4$ in the absence or presence of increasing concentrations of sodium pyruvate (Pyr) (2, 4, 6, 8 mM) for 24 h (a) or 48 h (b).

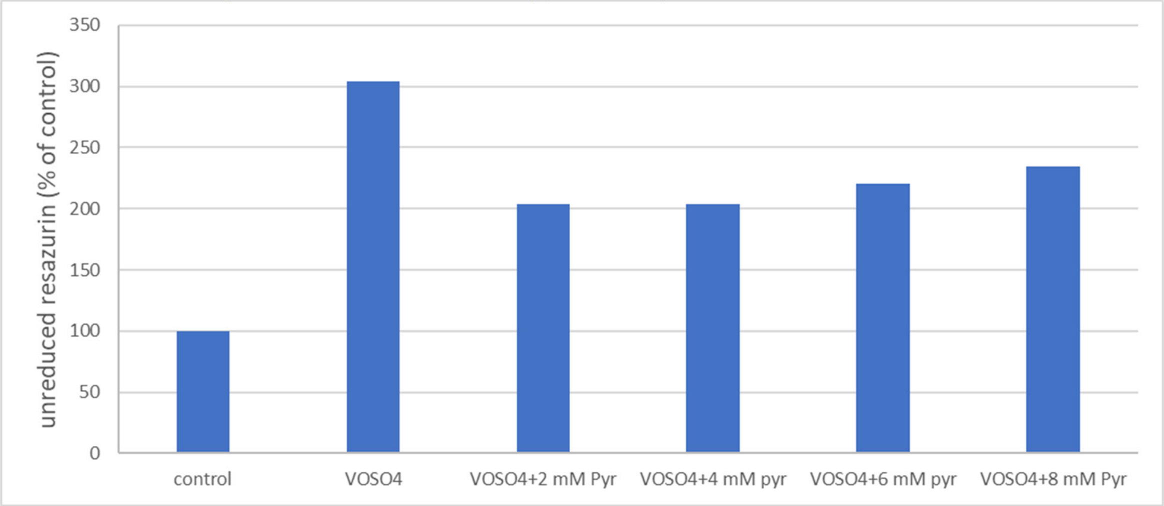

Figure S2. Effects of pyruvate on $VOSO_4$-induced cytotoxicity of CHO-K1 cells in the resazurin-based assay. The cells were incubated in DMEM/F12 medium without (control) or with 100 µM $VOSO_4$ in the absence or presence of increasing concentrations of sodium pyruvate (Pyr) (2, 4, 6, 8 mM) for 48 h. Data represent the mean ± SD of triplicate determinations of each data point,.
